#  Direct Interaction between AR and PAK6 in Androgen-Stimulated PAK6 Activation

**DOI:** 10.1371/journal.pone.0077367

**Published:** 2013-10-10

**Authors:** Xia Liu, Jennifer Busby, Ciny John, Jianning Wei, Xin Yuan, Michael L. Lu

**Affiliations:** 1 Urologic Research, Brigham and Women's Hospital, Department of Surgery, Harvard Medical School, Boston, Massachusetts, United States of America; 2 Department of Molecular Therapeutics, Scripps South Florida, Jupiter, Florida, United States of America; 3 Department of Biomedical Science, Florida Atlantic University, Boca Raton, Florida, United States of America; 4 Division of Hematology-Oncology, Beth Israel Deaconess Medical Center, Department of Medicine, Harvard Medical School, Boston, Massachusetts, United States of America; University of Kentucky College of Medicine, United States of America

## Abstract

A p21-activated kinase 6 (PAK6) was previously identified to be an androgen receptor (AR) interacting protein through a yeast two-hybrid screening. We used hormone responsive prostate cancer LAPC4 and LNCap cell lines as models to study the signaling events associated with androgen stimulation and PAK6. An androgen-stimulated PAK6 kinase activation was observed in LAPC4 cells expressing endogenous PAK6 and in LNCap cells ectopically expressing a wild type PAK6. This activation was likely mediated through a direct interaction between AR and PAK6 since siRNA knock-down of AR in LAPC4 cells downregulated androgen-stimulated PAK6 activation. In addition, LNCap cells expressing a non-AR-interacting PAK6 mutant exhibited dampened androgen-stimulated kinase activation. As a consequence of androgen-stimulated activation, PAK6 was phosphorylated at multiple serine/threonine residues including the AR-interacting domain of PAK6. Furthermore, androgen-stimulation promoted prostate cancer cell motility and invasion were demonstrated in LNCap cells ectopically expressing PAK6-WT. In contrast, LNCap expressing non-AR-interacting mutant PAK6 did not respond to androgen stimulation with increased cell motility and invasion. Our results demonstrate that androgen-stimulated PAK6 activation is mediated through a direct interaction between AR and PAK6 and PAK6 activation promotes prostate cancer cells motility and invasion.

## Introduction

The p21-activated kinases (PAKs) are a family of serine/threonine kinases sharing a conserved N-terminal Cdc42/Rac-interacting-binding domain (CRIB) and a C-terminal kinase catalytic domain [1,2]. Six members of this family, PAK 1-6, have been identified in mammalian cells thus far. Based on their sequence and structural similarities, PAKs have been categorized into Group 1 (PAK 1, 2 and 3) and Group 2 (PAK 4, 5 and 6) [3]. Group 1 PAK has a functional auto-inhibitory domain (AID) that flanks CRIB domain C-terminus to negatively regulate catalytic activity of the kinase. At resting stage, Group 1 PAKs exist as autoinhibited-dimers in which the catalytic activity is blocked by a direct interaction between the kinase domain and AID. Upon binding of p21 GTPase, GTP-Cdc42 or -Rac, to the CRIB domain, the AID blockade is released resulting in phosphorylation in the activation loop and subsequent kinase activation. The existence of a functional AID for Group 2 PAKs, PAK4 and PAK6, was demonstrated only recently [4,5]. Although the AIDs are somewhat conserved from both groups, it was demonstrated that the activation mechanism of Group 2 PAKs involves mainly the conformational change-induced ‘unlocking’ of the catalytic domain without an Activation-loop phosphorylation step [4,5]. 

Various cellular events, such as reorganizing cytoskeleton, modulating cell morphology, and promoting cell motility and cell survival, have been previously determined as downstream targets of Group 1 PAK, whereas much of the regulation and biological role of Group 2 PAK remains undefined. Many lines of evidence do suggest functional overlaps between these two groups. For example, PAK4 has been demonstrated to mediate cytoskeleton reorganization and filapodia formation in response to HGF stimulation [6,7]. Both PAK1 and PAK5 have been shown to regulate apoptosis by blocking proapoptotic BAD mitochondria translocation via phosphorylation of Serine 112 [8-10]. On the other hand, unlike Group 1 PAKs, Group 2 PAKs failed to rescue the Ste20 kinase mutant in a *S. cerevisiae* functional complementation test, suggesting divergent downstream targets between these Groups [8]. The division can be further illustrated by the less than ubiquitous tissue distribution of PAK5 and PAK6 as compared to the Group 1 PAKs [11]. As demonstrated by Northern blot analysis, PAK6 expression is highest in brain, testis, and prostate [1]. In prostate cancer cell lines, PAK6 expression is detected at higher levels in PC3, LAPC4, DU145 cells but not in LNCap cells [2]. While PAK6 does not appear to function directly as a cofactor in modulating androgen receptor (AR) transcriptional activity, the androgen-dependent interaction between PAK6 and AR suggest a potential crosstalk between these two pathways. 

In the present study, we demonstrate that androgen stimulation acts as an extracellular signal that activates PAK6 in an AR-dependent manner in prostate cancer LAPC4 and LNCap cells. The androgen-stimulated activation of PAK6 is accompanied by increased prostate cancer cell motility and invasion. Our results indicate that the interaction between PAK6 and AR is responsible, at least in part, for the activation of PAK6 in prostate cancer cells in response to androgen stimulation since knock-down of AR expression by AR-specific siRNA in LAPC4 cells downregulates androgen-stimulated PAK6 activation. LNCap cells expressing PAK6-WT, but not non-AR-interacting PAK6 mutant, respond to androgen stimulation with increased cell motility and matrigel invasion. 

## Materials and Methods

### Materials and Reagents

Histone H4, anti-actin monoclonal antibodies were purchased from Sigma (St. Louis, MO). Protein-A/G-conjugated Sepharose beads were from Amersham/Pharmacia (Piscataway, NJ). Monoclonal antibody 12CA5 against the hemaagglutinin (HA) tag was obtained from Berkeley Antibody (Berkeley, CA). Anti-PAK6 polyclonal anti-serum was custom generated against glutathione-S-transferase-PAK6 (#292-400) fusion peptide by Covance (Princeton, NJ). On-Target Smart Pool of AR-specific small interference RNA (Cat#: L-003400-00-0005) was obtained from Dharmacon (Waltham, MA). Matrigel was purchased from BD Bioscience (Franklin Lakes, NJ). Dihydrotestosterone (DHT) was obtained from Steraloids Inc (Newport, RI) 

### Expression Vectors and Constructs

The PAK6-5A and -5E mutants were generated by replacing wild type fragment (pcDNA3-PAK6-WT; Invitrogen, Carlsbad, CA) directly with synthetic mutant fragments respectively (Bio-Basic, Ontario). Expression vector pTRE (Clontech Laboratory, Mountain View, CA) was used for cloning of tetracycline inducible PAK6. For mammalian two-hybrid assays, the mammalian two-hybrid expression vectors expressing full length PAK6 and truncated mutants were constructed by cloning the corresponding fragments in-frame with partial herpes transactivating protein (VP16, residues 411–456) as a fusion gene into the pACT vector (Promega, Madison, WI). Full-length AR and truncated mutants were cloned in-frame fused with GAL4-DBD (residues 1–147) in a pBIND vector (Promega, Madison WI). Luciferase reporter pG5-Luc (luciferase driven by five tandem-repeats of Gal4 binding sequence-fused minimal promoter) (Promega) was used as the interaction responsive reporter and a pCMV-RL (renilla luciferase) was used as a control vector for monitoring transfection efficiency.

### Cell Culture and Transient Transfection

LNCap (CRL-1740) and PC3 (CRL-1435) cells were purchased from ATCC (Manassas, VA). LNCap and PC3 cells were grown in RPMI1640 (Invitrogen), supplemented with antibiotics and 10% fetal bovine serum (FBS). LAPC4 was a generous gift from Drs. Robert Reiter and Charles Sawyers [12]. LAPC4 cells were grown in Iscove’s Dulbecco's modified Eagle's medium (IMDM; high glucose Invitrogen)), supplemented with antibiotics and 10% FBS. Cells (6 x 10^5^) were transfected by electroporation with a total of 10 μg of plasmid DNA using a Gene Pulser from BioRad (Hercules, CA). 

### Gel Electrophoresis and Immunoblotting

Proteins were separated by SDS-PAGE with a standard reducing protocol. Following electrophoresis, proteins were electrotransferred to a nitrocellulose membrane. The protein bands were visualized by ponceau-S red staining. Blots were destained and blocked by 5% nonfat dry milk, 0.05% Tween 20, and 1% BSA in Tris-buffered saline [10 mM Tris (pH8.0), 135 mM NaCl]. Immunoblotting was performed with designated antibodies and visualized with an enhanced chemiluminescence detection system (ECL, Pierce, Supersignal, Rockford, IL) following the manufacturer's protocol. 

### Immunoprecipitation

Immunoprecipitation of PAK6 was performed following a standard protocol. In brief, cells were lysed in immunoprecipitation RIPA buffer containing 50 mM Tris (pH 7.4), 135 mM NaCl, 1% Triton X-100 (V/V), 0.25% deoxycholate (W/V), and 0.05% SDS (W/V) and supplemented with protease inhibitors (2 mM PMSF, 5 mM DIFP (Diisopropyl fluorophosphates), 5μg/ml pepstatin, 1 mM EDTA). Lysates (1mg total protein per IP reaction) were cleared by centrifugation at 12,000 x g for 20 min at 4°C. Supernatants were incubated with individual antibodies (1 μg) and protein A Sepharose beads (20 μl packed beads) at 4°C for 1 h. At the end of incubation, beads were washed 5 times with lysis buffer. The resulting immunoprecipitated immunocomplexes were solubilized in 40 μl of Laemmli sample buffer, proteins were separated using SDS-PAGE, and transferred to a nitrocellulose membrane. The protein complex was detected by western blot analysis and visualized on an X-ray film by exposure to ECL (Pierce, Supersignal). For phosphopeptide mapping by mass spectrometry, androgen-stimulated PAK6 was immunoprecipitated from LNCap-PAK6 cell lysate using anti-HA monoclonal antibody 12CA5 coupled to protein G Sepharose beads. Immunoprecipitated PAK6 was isolated by excising the corresponding PAK6 band from a SDS-PAGE gel. Phospho-peptides were identified by a core mass spectrometry facility in Scripps Florida. 

### In vitro kinase assay

Kinase reactions of immunoprecipitated PAK6 were performed in kinase buffer (50 mM HEPES, pH 7.4, 10 mM MgCl_2_, 2 mM MnCl_2_ and 2 mM DTT, 200 μM ATP) supplemented with 1.0 μg/reaction of histone H4 and 20 μCi/reaction of radioactive ATP. These reactions were incubated for 30 min at 30°C and stopped by addition of sample buffer containing SDS. The samples were subjected to SDS-PAGE, and ^32^P-labeled, phosphorylated proteins were visualized by autoradiograph. 

### Haptotactic transwell migration and Matrigel invasion assays

Haptotactic transwell cell migration assay was done using a modified Boyden chamber [13]. In brief, both sides of the transwell PET membrane were coated with fibronectin (0.5 μg/cm^2^ on the top side and 2.5 μg/cm^2^ on the bottom side). Prostate cancer LAPC4 cells were seeded inside the well at a density of 1 x 10^5^ cells/ml. Cells were let to migrate for 16-24 hr in a CO_2_ incubator. At the end of incubation, the transwells were removed and the un-migrated cells at top membrane were wiped away with a cotton swab. The migrated cells on the bottom surface were trypsinized and quantified using CyQuant NF (Invitrogen, Carlsbad, CA) following manufacturer’s instructions. 

The invasion assays were performed as previously described with some modifications [14]. In brief, 12-well transwell chambers (Costar, Cambridge, MA), 8 μm pore size, were coated with 300 μg/cm^2^ growth factor reduced BD-matrigel (BD Biosciences) by incubating at 37°C overnight. 4 x 10^4^ LNCap cells expressing PAK6-WT, PAK6-5A or PAK6-5E were mixed with matrigel diluted (300 μg/ml) in RPMI1640 (supplemented with 1% FBS) at 4°C then seeded on the matrigel coated transwell. Cell invasion was allowed for 16-24 hr at 37°C by adding 1.2 ml of complete media (RPMI1640/10% FBS) to the lower chamber of the transwell. At the end of the incubation, the non-invaded cells were scraped off using a cotton swap. The cells migrated across the membrane to the bottom surface of the PET membrane were stained with 1% crystal violet in methanol. Cell number was quantified by counting cells on the whole membrane microscopically. 

### Statistical analysis

All data are expressed as means ± S.E.M. To establish significance, group mean comparisons were performed with unpaired Student’s t-test or one-way ANOVA with post hoc Tukey test using GraphPad Prism 5.0 (San Diego, CA, USA). The criterion for significance was set at *p* ≤ 0.05.

## Results

### 1: PAK6 activation in response to androgen stimulation

**Figure 1:**
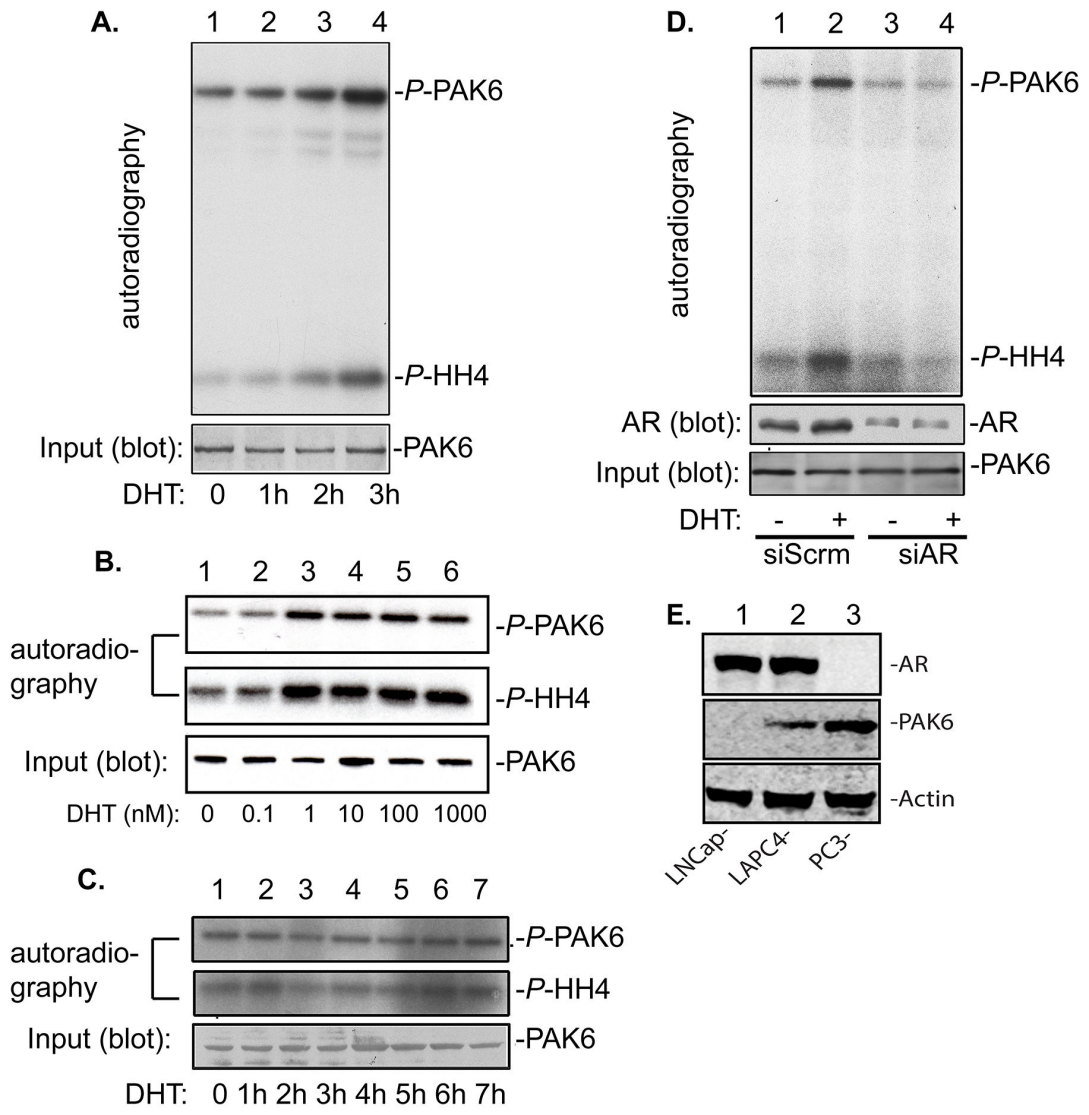
Androgen-stimulated PAK6 activation is mediated by androgen receptor. LAPC4 cells were plated overnight in IMDM supplemented with 10% charcoal-stripped fetal bovine serum. (A) Time course (0 h: non-treated) study of PAK6 activation in response to 10 nM dihydrotestosterone (DHT) stimulation in LAPC4 cells. (B) A DHT ten-fold serial dilution (0 nM: non-treated; and DHT-treated 0.1 nM to 1,000 nM) dose response of PAK6 activation. (C) 10 nM DHT does not activate PAK6 kinase activity in androgen receptor negative PC3-PAK6 cell. (D) PAK6 *in* *vitro* kinase assay demonstrates knocking down of AR by siRNA diminishes androgen-stimulated PAK6 activation in LAPC4 cells. Middle panels depict the expression levels of AR and PAK6 in LNCap-PAK6 cells in response to AR specific siRNA. (E) Whole lysate of LNCap, LAPC4 and PC3 blot against anti-PAK6,anti-AR and anti-actin.

Although PAK6 is identified as an AR interacting protein, it does not function as a co-factor in AR transcriptional activation [1]. To investigate the relationship between AR and PAK6, we first determined if PAK6 could be regulated via AR-mediated androgenic signals. To test this possibility, an AR-positive, PAK6-expressing prostate cancer LAPC4 cell line was used. LAPC4 cells were starved in serum free media overnight followed by stimulating with 10 nM of dihydrotestosterone (DHT) for various times before they were harvested for kinase assay. Androgen-stimulated PAK6 kinase activity was determined by an anti-PAK6 antibody immunoprecipitation-coupled *in vitro* kinase assay (IP-kinase assay). As shown in Figure 1A, in response to androgen stimulation, activation of PAK6 was evident by 1 hr and continue to be active at 3 hr post DHT stimulation. A dose response of PAK6 activation in response to various concentration of DHT, in a ten-fold serial dilution (from 0.1 nM to 1,000 nM), was demonstrated by an *in vitro* kinase assay (Figure 1B). However, in AR-negative PC3 cells, the PAK6 kinase activity remained unchanged in response to androgen stimulation throughout the time course with prolonged incubation (Figure 1C). To determine whether AR is required for DHT-stimulated activation of PAK6, LAPC4 cells were transfected with AR specific siRNA Smart-pool (Dharmacon) to downregulate AR expression levels. As shown in Figure 1D (middle panel), LAPC4 cells transfected with AR-specific siRNA (siAR) exhibited a reduced level of androgen receptor protein expression up to 80%. The AR protein levels remain high in cells transfected with non-silencing/sequence-scrambled control siRNA (siScrm). These cells were subjected to the same androgen stimulation coupled with a IP-kinase assay as described above. Shown in Figure 1C (upper panel), as a result of AR knock-down in LAPC4 cells, androgen treatment failed to activate PAK6 kinase activity as it did in non-knocking down control siRNA. The expression levels of AR and PAK6 in LNCap, LAPC4 and PC3 cell are shown in Figure 1E. Together, these results indicate that PAK6 activation in response to DHT stimulation is an AR-dependent event.

### 2: Mapping the PAK6 AR-interacting domain

**Figure 2:**
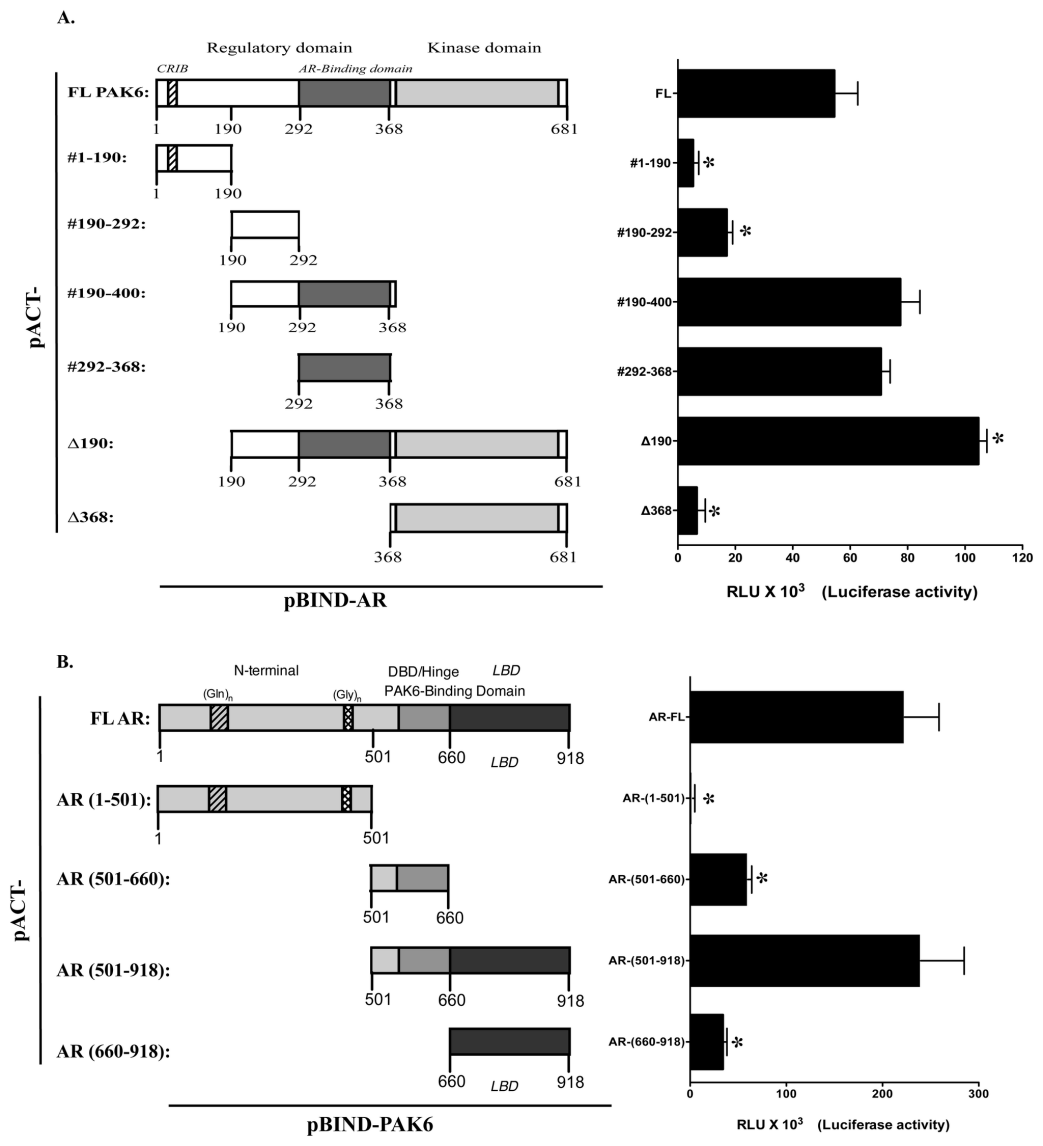
Mapping PAK6-AR interacting domain by mammalian two-hybrid assay. (A) Schematic depiction of full-length PAK6 and boundaries of truncated PAK6mutants encompassing various domains in the left panel. The right panel shows the results of mammalian two-hybrid assays. The interaction was determined by measuring the luciferase activities of HEK 293 cells transiently transfected with PAK6 full-length and deletion mutants fused in-frame downstream of VP16 transactivation domain in a pACT vector and GAL4 DBD fused AR in a pBIND vector. Data represent the mean ± SEM (n = 3). *p<0.05, compared to full-length PAK6, one-way ANOVA. (B) The left panel shows the schematic depiction of full-length AR and boundaries of AR truncated mutants encompassing various domains. A mammalian two-hybrid assay was performed by co-transfecting HEK-293cells with pACT-AR or truncated AR mutants, as indicated, with pBIND-PAK6 and pG5-Luc reporter construct. In both sets of experiment, a renilla luciferase reporter activity was used as a transfection internal control. The luciferase activity was normalized against the internal control and the vector basal control groups. Data represent the mean ± SEM (n = 3). *p<0.05, compared to full-length AR, one-way ANOVA.

Since PAK6 is identified as an androgen-dependent AR-interacting protein, we want to determine if the interaction per se is required for androgen simulated PAK6 activation. We mapped the domain/structural requirement of PAK6 responsible for interacting with AR using a mammalian two-hybrid assay. The mammalian two-hybrid assays were performed by co-transfecting the following expression vectors: a VP-16 transactivation domain fused with various deletion mutants of PAK6 (see Figure 2A for details), and a GAL4 DNA-binding domain fused with AR, and a pGal4-Luc reporter gene. The interactions were determined by an increased activity in a luciferase reporter assay. The results from the mammalian two-hybrid assay are shown in Figure 2A lower panel. Based on these results, an AR interacting domain of PAK6 is identified between residues #292-368 that localizes at the immediate upstream of the catalytic domain (Figure 2A). Coincidentally, the current identified AR-interacting domain overlaps with the PAK6 fragment originally identified through the yeast two-hybrid screening that spanning between residues #285-618 [1,2]. Reciprocally, the region of AR responsible for interacting with PAK6 was also identified by the same two-hybrid assay. As shown in Figure 2B, a positive interaction between PAK6 and AR DBD-hinge-LBD (DNA-binding, hinge and ligand-binding domain) (residues #501-918) was detected by this assay while DBD-hinge (residues #501-660) or LBD (residues #660-918) alone only exhibit partial interactive activities.

### 3: Identification of Phospho-Amino Acid Residues in Androgen Stimulation-Activated PAK6

**Figure 3:**
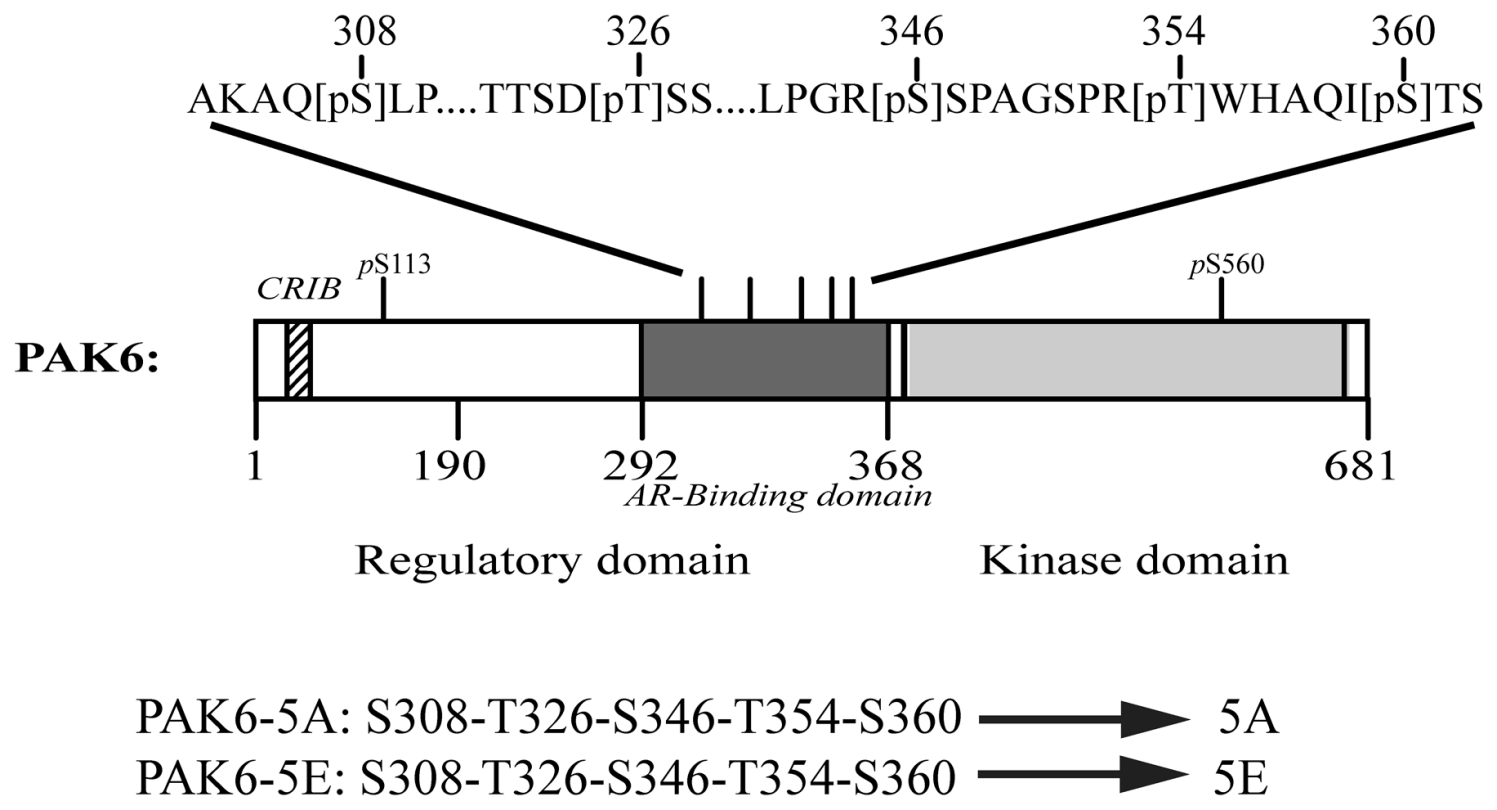
Schematic diagram of PAK6 AR-binding domain phosphorylation site mutations. The AR-binding domain constitutive non-phosphorylation mutant PAK6-5A is generated by substituting three serine and two threonine residues with alanine (A). The AR-binding domain constitutive phosphorylation mimic mutant PAK6-5E is generated by substituting Serine/Threonine residues with glutamic acid (E).

Next, we set out to identify the relevant amino acid residues of PAK6 within the AR-binding domain that actively participate in the regulation of PAK6 and AR interaction. We examined the sequence composition of the AR-binding domain of PAK6 to look for potential residues that may take part in the regulation of this interaction. We determined the possible phosphorylation site in immunoprecipitated androgen-activated PAK6 by mass spectrometry. As depicted in Figure 3, mass spectrometry analysis revealed multiple phospho-amino acids in PAK6 (Table 1 and the Fig S1-S6 in File S1) including one N-terminal peptide containing phospho-serine-113, one peptide containing phospho-serine-560 in the kinase domain and stretches of multiple phosphopeptides that localized within the ‘AR-binding domain’ of PAK6, including phospho-serines of residues: #308, #346 and #360; and phospho-threonine of residues: #326 and #354 (Figure 3, Table 1 and Figure S1-S6 in File S1). 

**Table 1:** PAK6 phospho-peptide sequences identified by mass spectrometry.

Residue #	Phospho-Peptide Sequence
#111-137	AQ[pS]LGLLGDEHWATDPDMXLQSPQSER
#306-331	AQ[pS]LPSDQPVGTFSPLTTSD[pTpSpS]PQK
#335-353	TAPATGQLPGR[pSpS]PAGSPR
#354-374	TWHAQI[pS]TSNLYLPQDPTVAK
#354-374	[pT]WHAQI[pS]TSNYLPQDPTVAK
#560-575	[pS]LVGTPYWMAPEVISR

### 4: Androgen-stimulated PAK6 activation requires direct interaction between AR and PAK6

**Figure 4:**
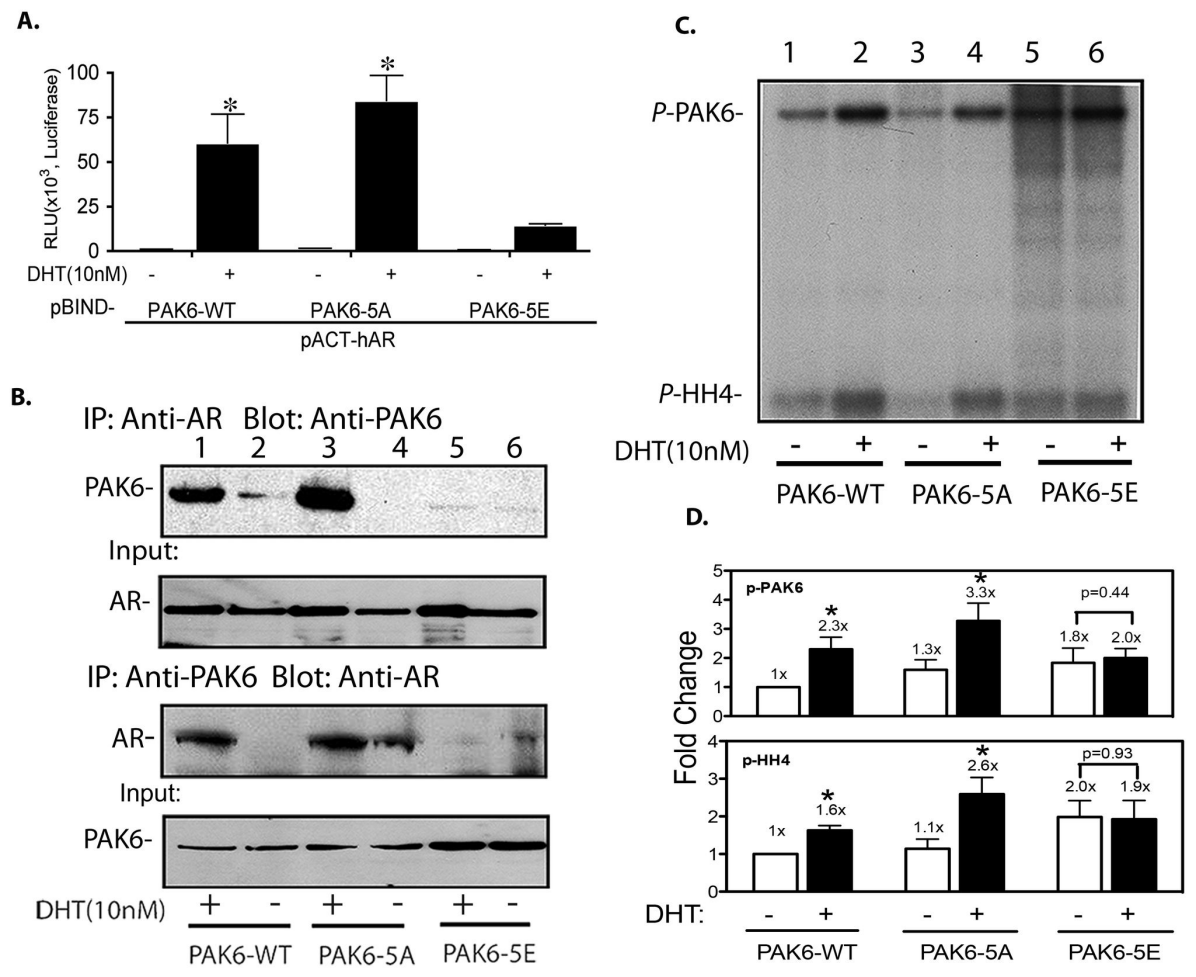
Phosphorylation site mutants of PAK6-5A and PAK6-5E exhibit altered ability to interact with AR and androgen-stimulated kinase activation. (A) Interactions between AR and PAK6-5A, PAK6-5E are assessed by a mammalian two-hybrid assay. Full-length AR is cloned in-frame downstream of VP16 transactivation domain with a HA-tag using a pACT vector. PAK6-wt, -5A and 5E were cloned in-frame downstream of GAL4 DBD with a HA tag using a pBIND vector. A mammalian two-hybrid assay was performed by co-transfecting HEK-293cells with pACT-AR or AR truncated mutants, as indicated, with pBIND-PAK6 and pG5-Luc vector. Cells were treated with vehicle control or DHT (10 nM) for 20 h followed by dual luciferase assay. A renilla luciferase reporter activity was used as a transfection internal control. The luciferase activity was normalized by the internal control and the vector basal control groups. Data represent the mean ± SEM (n=3, *p<0.01 compared to their respective vehicle controls; Student’s *t*-test). (B) A reciprocal immunoprecipitation assay was performed using AR and PAK6-WT, -5A and -5E mutants. HEK-293 cells were transfected with equal amount of AR and PAK6 expression vectors in the presence or absence of 10 nM DHT. Twenty hours post-transfection either AR or PAK6 was immunoprecipitated using antibodies specifically for either molecule then reciprocally probed by western blot for presence of co-precipitated interacting proteins as depicted. (C) A representative experiment of androgen-stimulated activation of PAK6-WT, PAK6-5A and PAK6-5E in an *in* *vitro* kinase assay. The upper phosphorylation band is PAK6 autophosphorylation. The lower bands are the phosphorylation of the exogenous substrate histone H4. (D) Quantification of relative kinase activities of in vitro kinase assays from (C). Data represent the mean ± SEM (n=4, *p<0.01 compared to their respective vehicle controls; Student’s *t*-test).

To test the potential involvement of AR-binding-domain phosphorylation in PAK6-AR interaction, we generated two PAK6 mutants that have those phosphorylation sites modified by either substituting the serine (at residues #308, 346 and 360) and threonine (residue #326 and 354) residues to alanine (S or T→A; PAK6-5A) to mimic "non-phosphorylated" state or to glutamic acid (S or T→E; PAK6-5E) to mimic "constitutive phosphorylated" state (Figure 3 and 4). The effects of these phosphorylation mimicking mutations on the interaction between PAK6 and AR were assessed by a mammalian two-hybrid assay. As shown in Figure 4A, the interaction between AR and PAK6-5A remained high as compared to PAK6-WT. In contrast, mutagenesis by substituting serines/threonine with glutamic acid, namely the phospho-mimetic PAK6-5E mutant, exhibited diminished androgen-dependent interaction between AR and PAK6 (Figure 4A). These interactions were further demonstrated through a reciprocal co-immunoprecipitation assay by co-transfection of AR and PAK6-WT, -5A or 5E. As shown in Figure 4B, an androgen-dependent interaction between AR and PAK6 is evident in PAK6-WT and PAK6-5A groups but no association was detected between AR and PAK6 phospho-mimicking mutant PAK6-5E. A minor weak interaction between AR and PAK6-5A was detectable in the anti-PAK6 immunoprecipitates even in the absence of androgen (Figure 4B, third panel lane 4). These results indicate that the interaction between PAK6 and AR is modulated by multi-phosphorylation of the PAK6 AR-binding-domain. 

Next, we determined if the interaction between AR and PAK6 is necessary for androgen-stimulated PAK6 activation. To this end, LNCap cells stably expressing PAK6-WT, PAK6-5A or PAK6-5E were established. The effects of these mutations on androgen-stimulated activation of PAK6 were determined by an immunoprecipitaion-coupled in vitro kinase assay. Figure 4C is shown as a representative experiment of the IP-kinase assay. The quantification of multiple experiments (n=5) of androgen-stimulated activation of PAK6-WT, -5A and -5E is shown in Figure 4D where PAK6-5A remained responsive to androgen stimulation-induced activation as its WT counterpart (*p*<0.01). On the other hand, there is a clear elevation of the baseline kinase activity of in the non-treated PAK6-5E group (Figure 4C and 4D) suggesting PAK6-5E mutant may be in the status of partial activation. The androgen stimulation has no effects on further promoting PAK6-5E mutant autophosphorylation or kinase activity toward the exogenous histone H4 substrate. The absence of androgen-stimulated kinase response of LNCap-PAK6-5E cells can potentially be attributed to the lack of interaction between AR and PAK6-5E. Taken together, our results support the notion that a physical interaction between AR and PAK6 is required in androgen-stimulated PAK6 activation. 

### 5: Activated PAK6 promotes prostate cancer cell motility

**Figure 5:**
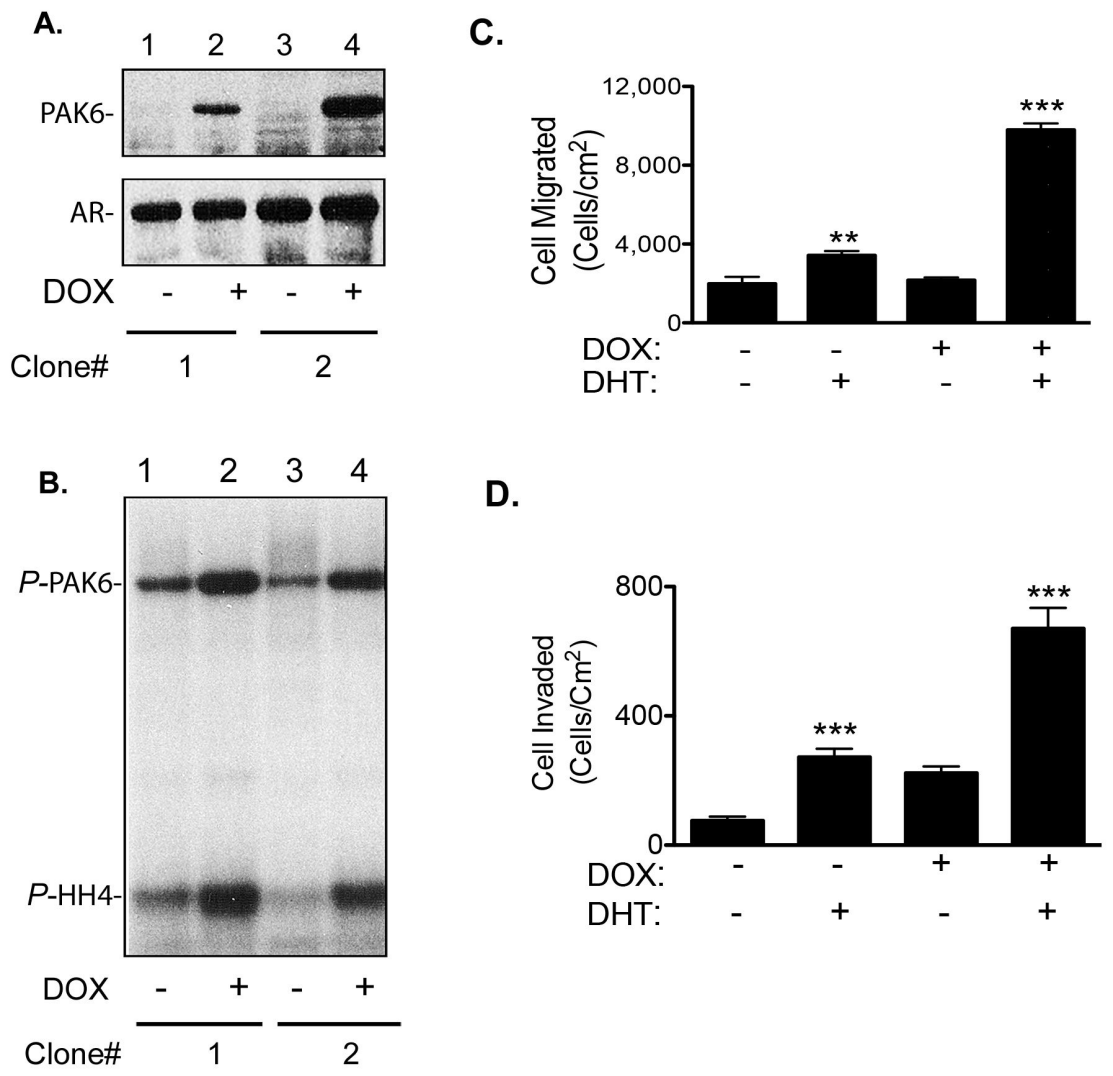
Androgen-stimulated PAK6 activation promotes cell motility and invasion in LNCap cells stably transfected with tetracycline-inducible PAK6 vector (LNCap-tetON-PAK6). (A) Anti-PAK6 immuno-blot of tetracycline inducible PAK6 expression in two different clones of LNCap cells depicts the induction of PAK6 expression by doxycycline (1 μg/ml). (B) In vitro kinase activities of doxycycline-induced PAK6 kinase from individual clones using histone H4 as an exogenous substrate. (C) Androgen stimulation promotes transwell cell migration of doxycycline-induced LNCap-tetOn-PAK6 cells. Data represent the mean ± SEM. (n=6). **p<0.01, ***p<0.001 compared to their respective –DHT control; Student’s *t*-test. (D) Androgen stimulation promotes matrigel invasion of doxycycline-induced LNCap-tetON-PAK6 cells. All experiments were repeated at least three times, with consistent results. Data represent the mean ± SEM. (n=6). ***p<0.001 compared to their respective –DHT control; Student’s *t*-test .

To characterize the physiological consequences of androgen-stimulated PAK6 activation, we established LNCap cell lines (LNCap-tetON-PAK6) ectopically expressing PAK6 WT under the control of a tetracycline-inducible promoter. The inducible PAK6 LNCap cell lines allowed us to evaluate the event of androgen stimulation and PAK6 activation in a homogeneous clonal cell background. As shown in Figure 5A, tetracycline-dependent inductions of PAK6 expression are demonstrated in two representative LNCap-tetON-PAK6 clones while expression of AR is not affected by the treatment. In an in vitro kinase assay, the tetracycline-induced PAK6 responds to androgen stimulation with increased kinase activity towards exogenous substrate histone H4 (Figure 5B, lower bands) and an elevated autophosphorylation (Figure 5B, upper bands). Although no PAK6 expression was detected in the western blot of non-induced groups (Figure 5A lanes 1 and 3 upper panel), minor PAK6 autophosphrylation bands are visible (Figure 5B, lanes 1 and 3 upper bands) suggested a low level leakiness of the tetracycline inducible promoter.

To illustrate the effects of androgen-stimulated PAK6 activation in regulating cell motility, a transwell haptotatic migration assay using a modified Boyden chamber was performed as described previously [15]. As shown in Figure 5C, the non-induced LNCap-tetON-PAK6 cells exhibit baseline motility in the absence of androgen while the tetracycline-induced androgen-stimulated LN-tetON-PAK6 cells exhibit highest migratory motility (*p*<0.001). A moderate increase of cell migration is also observed in the presence of androgen without tetracycline induction (*p*<0.01) that may result from the expression of a low level PAK6 due to promoter leakiness. No promotion of migration can be observed in cells induced to express PAK6 without androgen stimulation. 

A matrigel invasion assay was performed to determine the potential effects of PAK6 activation on modulating the invasive ability of LNCap. As shown in Figure 5D, androgen stimulation dramatically promotes matrigel invasion (*p*<0.001) of tetracycline-induced LNCap-tetON-PAK6 cells as compared to non-induced cells. A less prominent effect of androgen-stimulation promoted invasion is also evident in non-induced LNCap cells presumably due to a leaky PAK6 expression. These results indicate androgen-stimulated PAK6 activation promotes LNCap cells haptotactic migration and matrigel invasion. 

**Figure 6:**
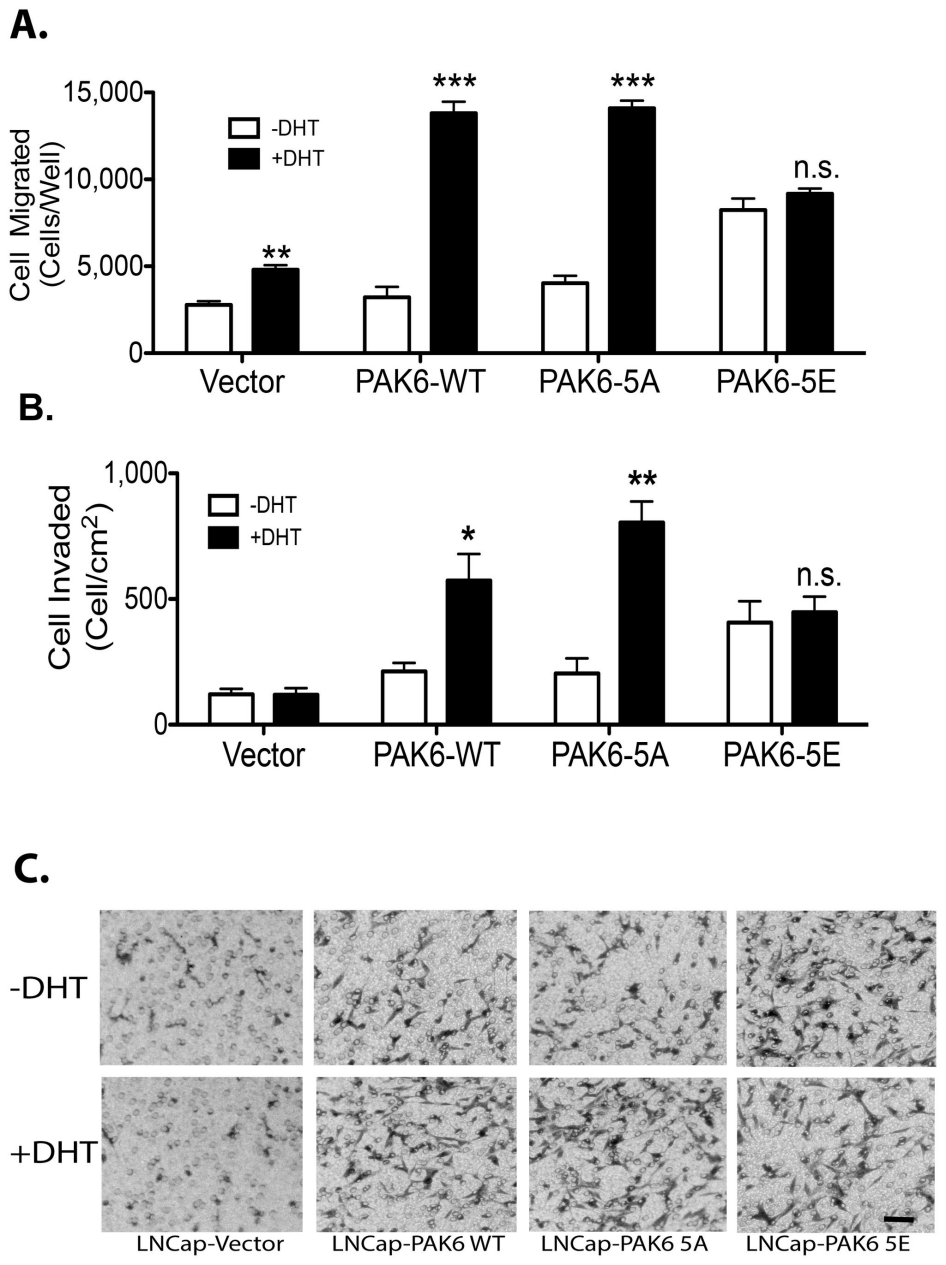
Androgen-stimulated PAK6 activation promotes cell motility and invasion in LNCap cells stably expressing PAK6-WT, PAK6-5A and PAK6-5E. (A) Androgen stimulation promotes transwell cell migration of LNCap-PAK6-WT and LNCap-PAK6-5A cells. LNCap-PAK6-5E cells, albeit with elevated baseline motility, do not respond to androgen stimulation with increased migration. Data represent the mean ± SEM (n=3). **p<0.01, ***p<0.001 compared to their respective –DHT controls; Student’s *t*-test. (B) Androgen stimulation promotes matrigel invasion of LNCap PAK6-WT and LNCap-PAK6-5 cells. Similarly, LNCap-PAK6-5E cells, albeit with elevated baseline invasion, do not respond to androgen stimulation with increased invasion. Data represent the mean ± SEM (n=3). *p<0.05, **p<0.01 compared to their respective –DHT controls; Student’s *t*-test. (C) Representative micrographs of invaded LNCap and PAK6-derivertives cells depicted in (B). Scale bar: 50 μm.

To further examine the role of AR-PAK6 interaction played in promoting the androgen-stimulated cell motility and invasion, we established LNCap cell lines stably expressing PAK6 WT, -5A or -5E. The same haptotatic assay and matrigel invasion assay were employed for this purpose. As shown in Figure 6A, androgen stimulation promoted-cell migration was observed in LNCap cells expressing either PAK-WT or PAK6-5A (*p*<0.001). Consistent with an elevated baseline kinase activity of PAK6-5E as observed in previous section (Figure 4C and 4D), a higher baseline motility was also observed in LNCap-5E cells in the absence of androgen stimulation. Consequently, the LNCap cells expressing PAK6-5E did not respond to androgen stimulation with further increase of cell motility

A matrigel invasion assay was performed to determine the potential effects of PAK6 activation on modulating the invasive ability of LNCap expressing various mutants of PAK6. As shown in Figure 6B, androgen stimulation dramatically promotes matrigel invasion in LNCap-PAK6-WT and LNCap-PAK6-5A cells (*p*<0.001). Representative micrographs of the invaded cells from each group are shown in Figure 6C. Similar to the observation described above, with an already elevated baseline invasion, the LNCap-5E exhibited no further upregulation of androgen-stimulated invasion. These observations are consistent with the notion that a direct interaction between AR and PAK6 is required for the kinase activation, thereafter, promotion of cell motility and invasion. 

## Discussion

In current study, we present evidence linking androgen and PAK6 activation to prostate cancer cell motility and invasion. We demonstrated that androgen stimulation acts as an extracellular signal that activates PAK6 in an androgen receptor-dependent manner in prostate cancer LAPC4 and LNCap cells. The androgen-stimulated activation of PAK6 is accompanied by multiple serine/threonine phosphorylation as determined by mass spectrometry. We showed that the interaction between PAK6 and AR is regulated by the PAK6’s AR-binding domain phosphorylation. Our data indicate that a direct interaction between AR and PAK6 is necessary for the activation of PAK6 in response to androgen stimulation. Consequently, androgen-stimulated PAK6 activation promotes prostate cancer cell motility and invasion. 

Our finding of androgen as an extracellular stimulus for PAK6 activation represents a novel androgen-dependent AR non-genomic signal pathway. AR can form a ternary membrane-associated signal complex with c-Src, estrogen receptor, and function cooperatively to activate Src kinase activity [16-18] to stimulate prostate cancer cell growth in a non-transcription-dependent manner. AR is also demonstrated to signal from plasma membrane lipid raft, a cholesterol rich sub-domain, to activate Akt or Erk [19-21] that promote cell growth and survival. Our data indicate that androgen-stimulated PAK6 activation requires a direct interaction between PAK6 and AR. While the exact mechanism of this activation process remains to be further characterized, two recent reports [4,5] describe the identification of a N-terminal localized pseudosubstrate-like autoinhibitory domain in group 2 PAKs may offer insights to a potential mechanism of our observations. In those reports, an intra-molecular interaction between the auto-inhibitory domain and the catalytic domain was determined to be responsible for keeping the group 2PAKs inactive, whilst interruption of this interaction activates it. The AR-stimulated phosphorylation of PAK6’s AR-binding domain, as described in the current study, may result in a conformational change that consequently activates PAK6 by perturbing the interaction between auto-inhibitory domain and catalytic domain. This possibility is further echoed by our result that the ‘phosphorylation-mimicking’ mutant PAK6-5E exhibits higher basal kinase activity in an in vitro kinase assay (Figure 4C and 4D). 

The AR-binding domain (residues 292-368) identified in current study is included in and as part of the original yeast two-hybrid screening identified AR-interacting PAK6 fragment (residues 285-681) [1]. An FxxMF motif located between residues #261-265 of PAK6 was recently described to bind AR [22]. Notably, the FxxMF motif is not part of the original PAK6 fragment identified by the yeast two-hybrid screening [22]. This suggests that the FxxMF motif may cooperative with additional structural components of PAK6 to mediate the interaction between PAK6 and AR. It is conceivable that additional ‘regulatory domain(s)’ exists in PAK6 that also partakes in the interaction between AR and PAK6. As shown in Figure 2A, the interaction between AR and PAK6 Δ190 deletion mutant appears to be stronger than the interaction between AR and full-length PAK6 in the mammalian two hybrid assay. This suggests a potential inhibitory domain, such as the ‘auto-inhibitory domain’ described above, may be present in amino acids #1-#190 of PAK6 that participates in the regulation of the PAK6-AR interaction. Moreover, our data demonstrate that phosphorylation of AR-binding domain profoundly influences the interaction between PAK6 and AR. Although the identity of the kinase(s) responsible for this phosphorylation is yet to be determined, our results underscore the potential complexity of the multistep nature of androgen-stimulated PAK6 activation. As evident from mammalian two-hybrid assay results depicted in Figures 2 and 4A, it is plausible that the PAK6-binding domain of AR only becomes accessible to interact with PAK6 upon androgen ligation. Once bound with AR, PAK6 may undergo autophosphorylation or be phosphorylated by an additional kinase(s) yet to be identified. Phosphorylation of AR-binding domain disengages PAK6 from its AR association that eventually results in full kinase activation. As for mapping of AR, the PAK6-binding domain of AR was previously determined to localize at the AR ligand-binding domain (LBD) excluding the AR DNA-binding domain (DBD) by an GST pull-down assay *in vitro* [2]. In the current mammalian two-hybrid assay, we show that both DBD and LBD of AR are required for a strong interaction between these two molecules *in vivo* since DBD or LBD alone exhibits only moderate PAK6-interacting activities. Nonetheless, these two seemingly different observations are not mutually exclusive; rather it highlights the limitations of different techniques used to determine the interactions. 

In conclusion, the finding that androgen stimulation promotes cell motility and invasion via activation of PAK6 is significant. This suggests a potential role of PAK6 in prostate cancer metastatic progression. It also offers a plausible mechanism for the previously reported [23] positive correlations between PAK6 expression and high-grade prostate cancer Since steroid hormone such as androgen has long been recognized in promoting prostate cancer progression, our results provide a direct molecular link between androgenic signal and metastasis related molecular events. 

## Supporting Information

File S1 **Supporting Figures S1-S6.**
**Figure S1.** Identification of Serine-308 as a site of phosphorylation. The amino acid sequence is provided above the spectrum, and the masses above and below the sequence correspond to the theoretical b- and y-type product ions, respectively. The masses provided are the singly-protonated, monoisotopic product ion masses. The observed singly-protonated product ions are underlined. For simplicity, all doubly protonated ions are not labeled in the spectra as they exist at 50% abundance or less. Asterisks indicate ions that result from neutral loss of H3PO4.
**Figure S2.** Identification of Threonine-326 as a site of phosphorylation. The amino acid sequence is provided above the spectrum, and the masses above and below the sequence correspond to the theoretical b- and y-type product ions, respectively. The masses provided are the singly-protonated, monoisotopic product ion masses. The observed singly-protonated product ions are underlined. Asterisks indicate ions that result from neutral loss of H3PO4 from fragment ions. Additionally, the presence of the second site of phosphorylation is determined due to accurate mass of the peptide, however, the first fragment ion representing this site is located at y7, thus the phosphorylation could exist in any of the sites shown in brackets.
**Figure S3.** Identification of Serine-346 as a site of phosphorylation. The amino acid sequence is provided above the spectrum, and the masses above and below the sequence correspond to the theoretical b- and y-type product ions, respectively. The masses provided are the singly-protonated, monoisotopic product ion masses. The observed singly-protonated product ions are underlined. The site of phosphorylation cannot be definetively identified, due to lack of specific ions related to either site of phosphorylation, however we hypothesize that the phosphorylation is on the first of the serine residues due to lack of tryptic cleavage at the preceding site. Asterisks indicate ions that result from neutral loss of H3PO4.
**Figure S4.** Identification of Thrionine-354 and Serine-360 as sites of phosphorylation. The amino acid sequence is provided above the spectrum, and the masses above and below the sequence correspond to the theoretical b- and y-type product ions, respectively. The masses provided are the singly-protonated, monoisotopic product ion masses. The observed singly-protonated product ions are underlined. Asterisks indicate ions that result from neutral loss of H3PO4 from fragment ions. This doubly phosphorylated peptide loses two phosphoric acid groups from the parent ion which are marked with *.
**Figure S5.** Identification of Serine-360 as a site of phosphorylation. The amino acid sequence is provided above the spectrum, and the masses above and below the sequence correspond to the theoretical b- and y-type product ions, respectively. The masses provided are the singly-protonated, monoisotopic product ion masses. The observed singly-protonated product ions are underlined. Asterisks indicate ions that result from neutral loss of H3PO4 from fragment ions.
**Figure S6.** Identification of Serine560 as a site of phosphorylation. The amino acid sequence is provided above the spectrum, and the masses above and below the sequence correspond to the theoretical b- and y-type product ions, respectively. The masses provided are the singly-protonated, monoisotopic product ion masses. The observed singly-protonated product ions are underlined. Asterisks indicate ions that result from neutral loss of H3PO4.
(PDF)
