# Supplementary material for: Direct Interaction between AR and PAK6 in Androgen-Stimulated PAK6 Activation
Source: PLoS One. 2013 Oct 10;8(10):e77367. doi: 10.1371/journal.pone.0077367 (PMC3795072; doi:10.1371/journal.pone.0077367)
Supplement: File S1 — Supporting Figures S1-S6. Figure S1. Identification of Serine-308 as a site of phosphorylation. The amino acid sequence is provided above the spectrum, and the masses above and below the sequence correspond to the theoretical b- and y-type product ions, respectively. The masses provided are the singly-protonated, monoisotopic product ion masses. The observed singly-protonated product ions are underlined. For simplicity, all doubly protonated ions are not labeled in the spectra as they exist at 50% abundance or less. Asterisks indicate ions that result from neutral loss of H3PO4. Figure S2. Identification of Threonine-326 as a site of phosphorylation. The amino acid sequence is provided above the spectrum, and the masses above and below the sequence correspond to the theoretical b- and y-type product ions, respectively. The masses provided are the singly-protonated, monoisotopic product ion masses. The observed singly-protonated product ions are underlined. Asterisks indicate ions that result from neutral loss of H3PO4 from fragment ions. Additionally, the presence of the second site of phosphorylation is determined due to accurate mass of the peptide, however, the first fragment ion representing this site is located at y7, thus the phosphorylation could exist in any of the sites shown in brackets. Figure S3. Identification of Serine-346 as a site of phosphorylation. The amino acid sequence is provided above the spectrum, and the masses above and below the sequence correspond to the theoretical b- and y-type product ions, respectively. The masses provided are the singly-protonated, monoisotopic product ion masses. The observed singly-protonated product ions are underlined. The site of phosphorylation cannot be definetively identified, due to lack of specific ions related to either site of phosphorylation, however we hypothesize that the phosphorylation is on the first of the serine residues due to lack of tryptic cleavage at the preceding site. Asterisks indicate ion [file pone.0077367.s001.pdf]

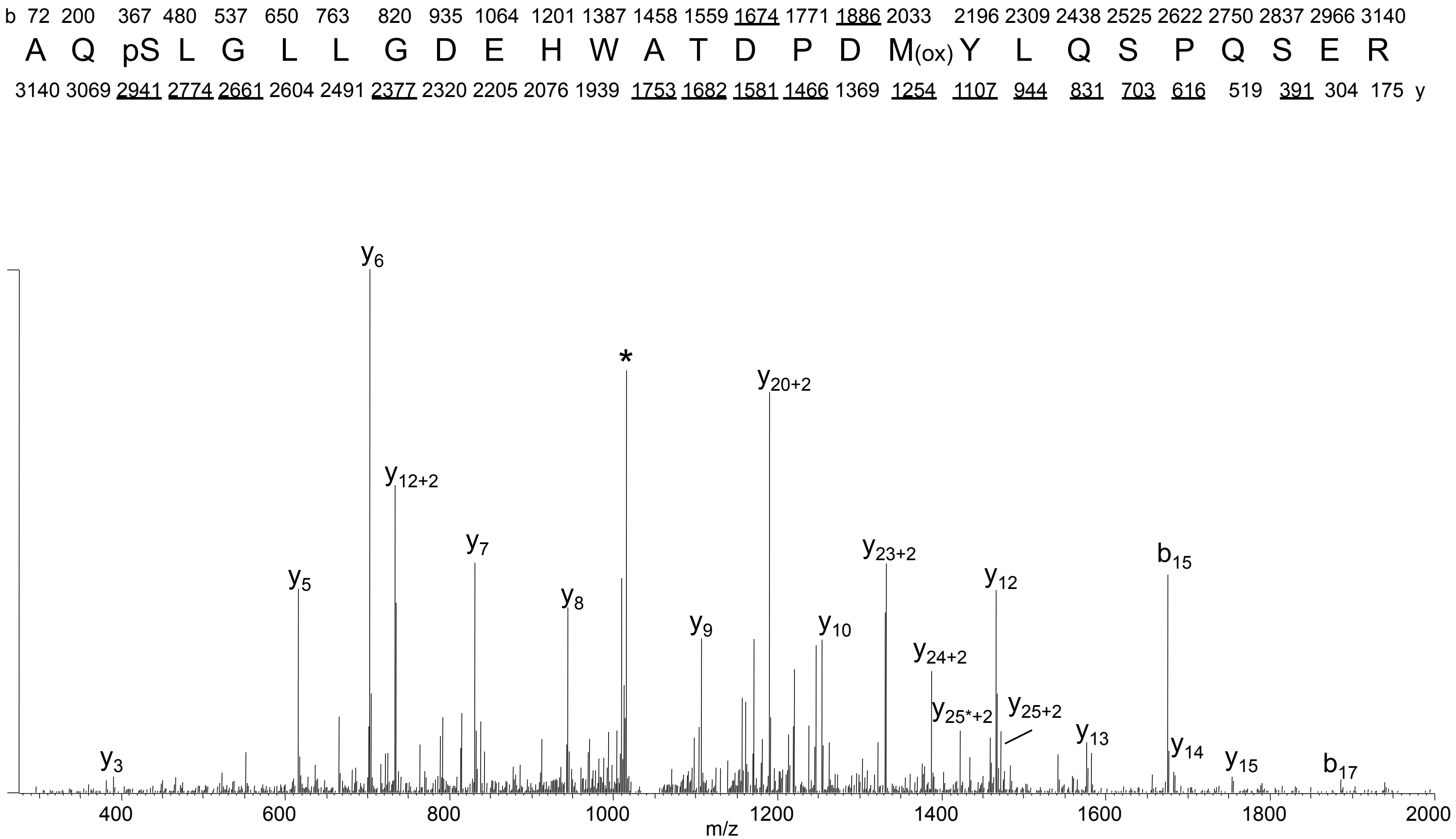

Supplemental Figure S1. Identification of Serine-308 as a site of phosphorylation.
The amino acid sequence is provided above the spectrum, and the masses above and below the sequence correspond to the theoretical b- and y-type product ions, respectively. The masses provided are the singly-protonated, monoisotopic product ion masses. The observed singly-protonated product ions are underlined. For simplicity, all doubly protonated ions are not labeled in the spectra as they exist at 50% abundance or less. Asterisks indicate ions that result from neutral loss of H_3PO_4.

b 72 200 367 480 577 664 779 907 1004 1103 1160 1261 1408 1495 1592 1705 1806 1907 1994 2109 2290 2377 2465 2562 2690 2836

A Q pS L P S D Q P V G T F S P L T T S D p(T S S) P Q K

2836 2765 2637 2470 2357 2260 2172 2057 1929 1832 1733 1676 1575 1428 1341 1244 1131 1030 929 842 727 546 459 372 275 147 y

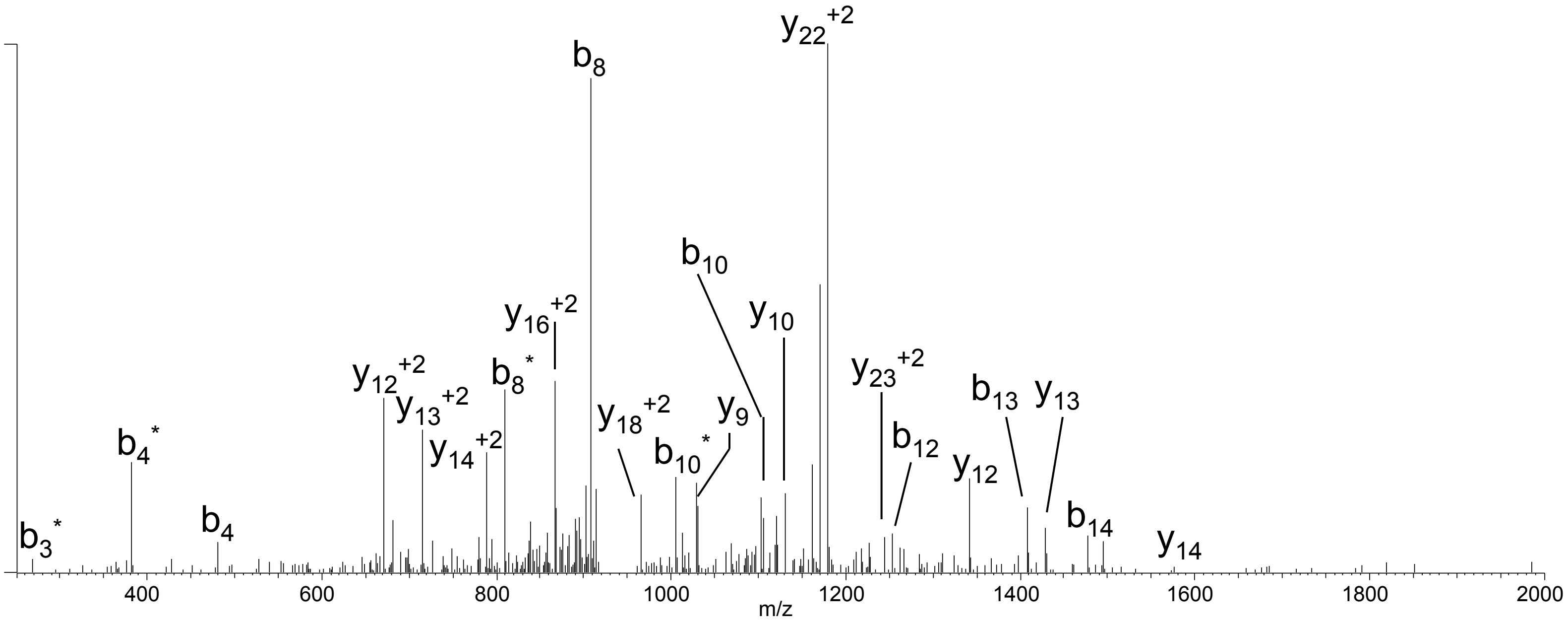

Supplemental Figure S2. Identification of Threonine-326 as a site of phosphorylation.
The amino acid sequence is provided above the spectrum, and the masses above and below the sequence correspond to the theoretical b- and y-type product ions, respectively. The masses provided are the singly-protonated, monoisotopic product ion masses. The observed singly-protonated product ions are underlined. Asterisks indicate ions that result from neutral loss of H_3PO_4 from fragment ions. Additionally, the presence of the 2nd site of phosphorylation is determined due to accurate mass of the peptide, however, the first fragment ion representing this site is located at y7, thus the phosphorylation could exist in any of the sites shown in brackets.

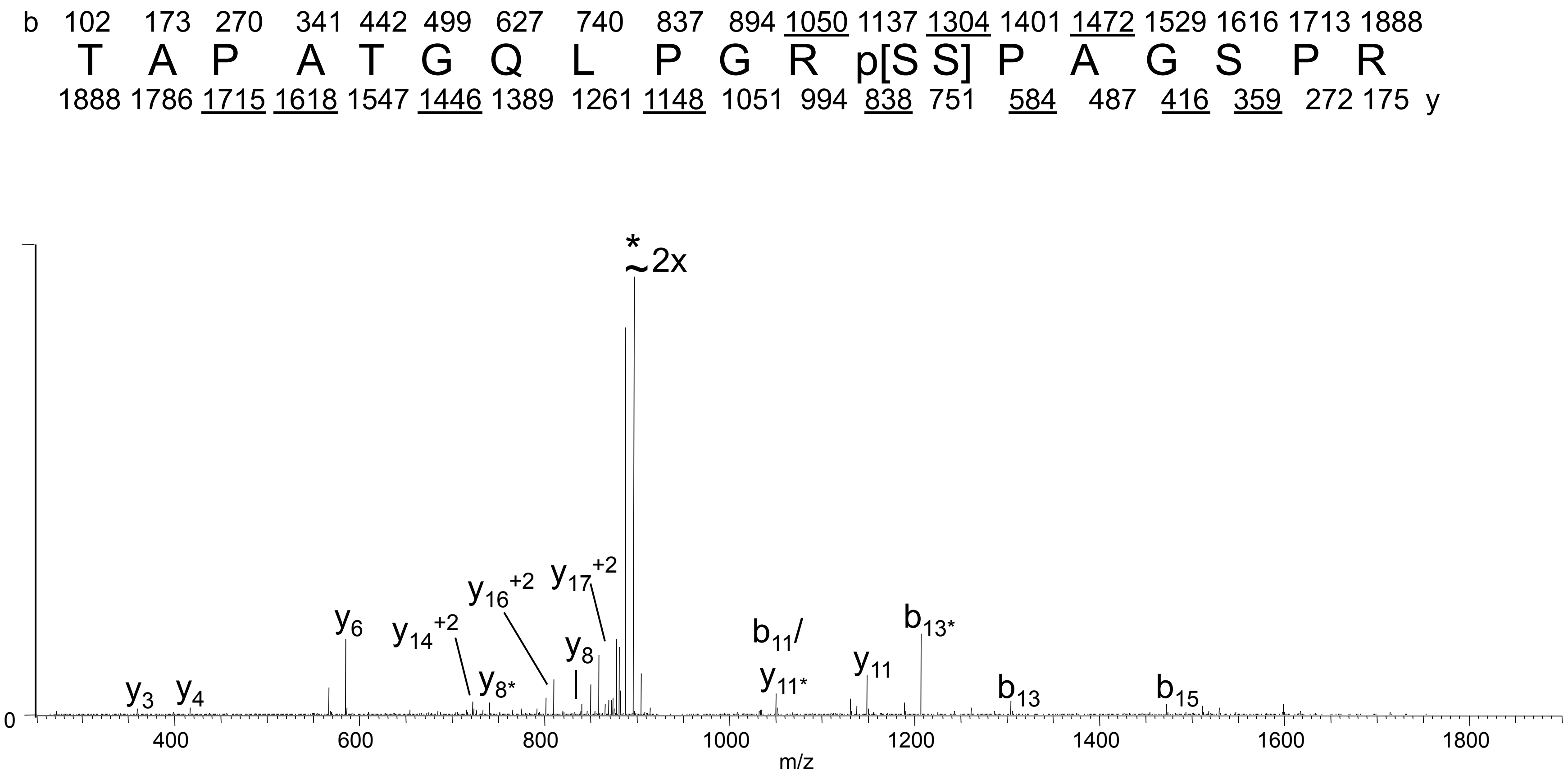

Supplemental Figure S3. Identification of Serine-346 as a site of phosphorylation.
The amino acid sequence is provided above the spectrum, and the masses above and below the sequence correspond to the theoretical b- and y-type product ions, respectively. The masses provided are the singly-protonated, monoisotopic product ion masses. The observed singly-protonated product ions are underlined. The site of phosphorylation cannot be definetively identified, due to lack of specific ions related to either site of phosphorylation, however we hypothesize that the phosphorylation is on the first of the serine residues due to lack of tryptic cleavage at the preceding site. Asterisks indicate ions that result from neutral loss of H_3PO_4.

b 182 368 505 576 704 817 984 1085 1172 1286 1399 1562 1675 1772 1900 2015 2112 2213 2312 2384 2530

pT W H A Q I pS T S N L Y L P Q D P T V A K

2530 2349 2163 2025 1954 1826 1713 1546 1445 1358 1244 1131 968 855 758 630 515 418 317 218 147 y

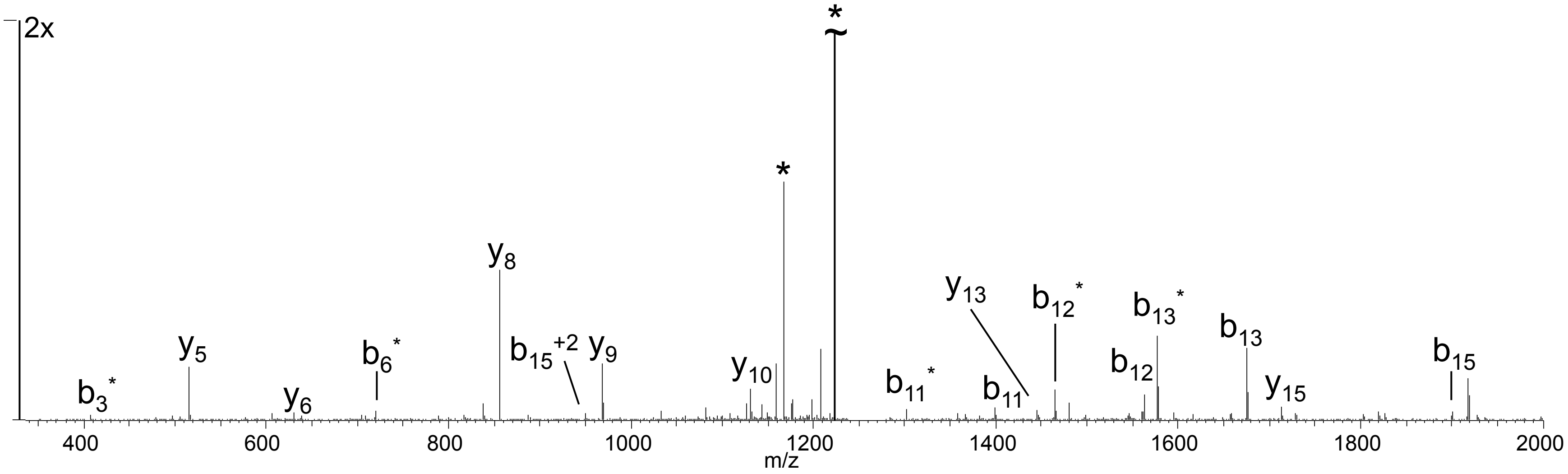

Supplemental Figure S4. Identification of Thrionine-354and Serine-360 as a sites of phosphorylation.
The amino acid sequence is provided above the spectrum, and the masses above and below the sequence correspond to the theoretical b- and y-type product ions, respectively. The masses provided are the singly-protonated, monoisotopic product ion masses. The observed singly-protonated product ions are underlined. Asterisks indicate ions that result from neutral loss of H_3PO_4 from fragment ions. This doubly phosphorylated peptide loses two phosphoric acid groups from the parent ion which are marked with *.

b	102	288	425	496	624	737	904	1005	1092	1206	1319	1482	1595	1692	1820	1935	2032	2133	2233	2304	2450
	T	W	H	A	Q	I	pS	T	S	N	L	Y	L	P	Q	D	P	T	V	A	K
y	2450	2349	2163	2025	1954	1826	1713	1546	1445	1358	1244	1131	968	855	758	630	515	418	317	218	147

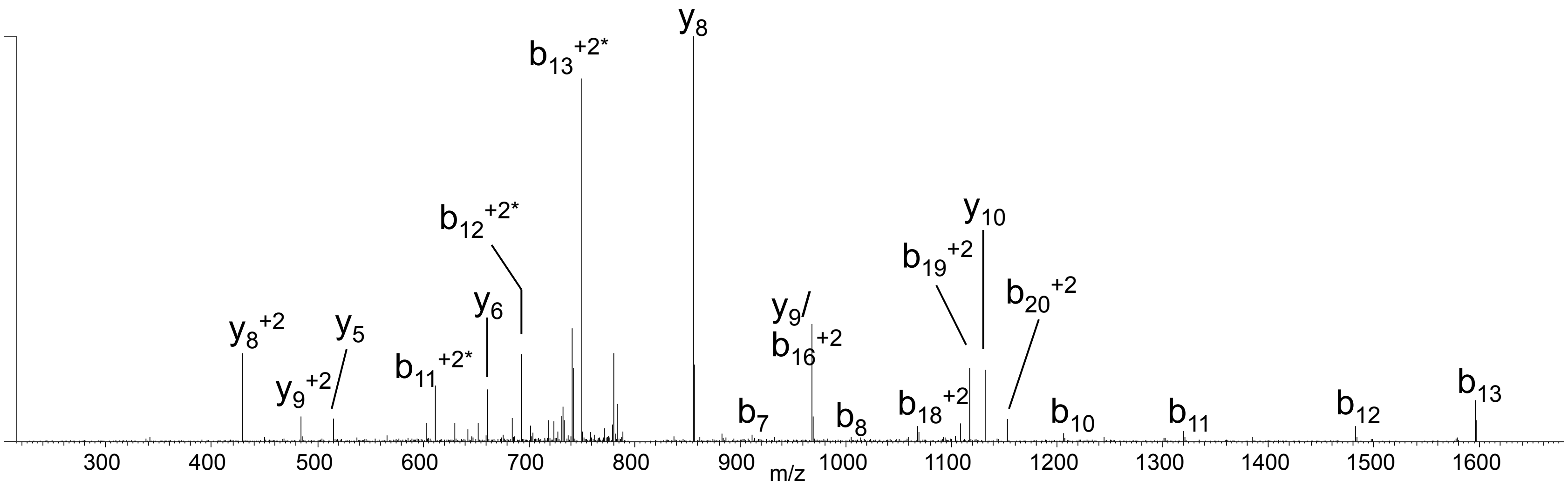

Supplemental Figure S5. Identification of Serine-360 as a site of phosphorylation.
The amino acid sequence is provided above the spectrum, and the masses above and below the sequence correspond to the theoretical b- and y-type product ions, respectively. The masses provided are the singly-protonated, monoisotopic product ion masses. The observed singly-protonated product ions are underlined. Asterisks indicate ions that result from neutral loss of H_3PO_4 from fragment ions.

b	168	281	380	437	538	635	798	984	1131	1202	1299	1428	1527	1640	1727	1902
	pS	L	V	G	T	P	Y	W	M(ox)	A	P	E	V	I	S	R
y	1902	1734	1621	1522	1465	1364	1267	1104	918	771	700	603	474	375	262	175

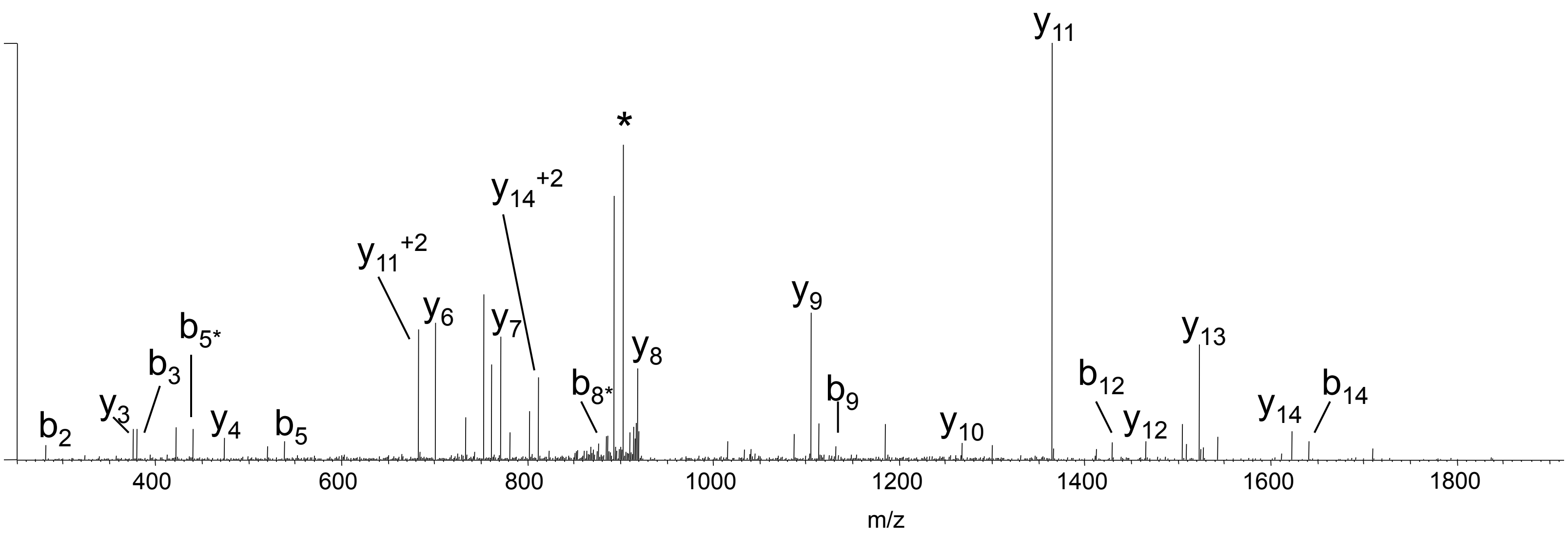

Supplemental Figure S6. Identification of Serine560 as a site of phosphorylation.
The amino acid sequence is provided above the spectrum, and the masses above and below the sequence correspond to the theoretical b- and y-type product ions, respectively. The masses provided are the singly-protonated, monoisotopic product ion masses. The observed singly-protonated product ions are underlined. Asterisks indicate ions that result from neutral loss of H_3PO_4.
